# Telechemistry 2.0: Remote monitoring of fluorescent chemical reactions

**DOI:** 10.1016/j.ohx.2021.e00244

**Published:** 2021-10-30

**Authors:** Chun-Yao Hsu, Gurpur Rakesh D. Prabhu, Pawel L. Urban

**Affiliations:** a Department of Chemistry, National Tsing Hua University, 101, Section 2, Kuang-Fu Rd., Hsinchu 30013, Taiwan; b Frontier Research Center on Fundamental and Applied Sciences of Matters, National Tsing Hua University, 101, Section 2, Kuang-Fu Rd., Hsinchu 30013, Taiwan

**Keywords:** Fluorescence detection, Internet-of-Chemical-Things, Reaction monitoring, Sensors

## Abstract

Implementation of the Internet-of-Things in chemistry research has the potential to improve research methodologies. Here, we describe a cloud-integrated real-time laboratory monitoring system for: (i) monitoring reactions involving fluorescent chemical species, and (ii) monitoring laboratory environment for safety purpose. A probe-type fluorescence detection system has been constructed to monitor reactions that involve fluorescent molecules. This device incorporates an in-house-built 3D-printed probe, two optical fibers, a light-emitting diode, a photoresistor, and a microcontroller board (MCB). The MCB relays experimental data to a single-board computer (SBC), which then uploads the data to a cloud-based platform (*ThingSpeak*) for data storage and visualization. The SBC is also connected to auxiliary sensors to measure relative alcohol vapor concentration, temperature, and humidity at different locations in the laboratory. The device has been validated and tested for its performance by monitoring a fluorescent chemical reaction (synthesis of fluorescent gold nanoclusters) for a period of 12 h.

## Graphical abstract

**Figure:**
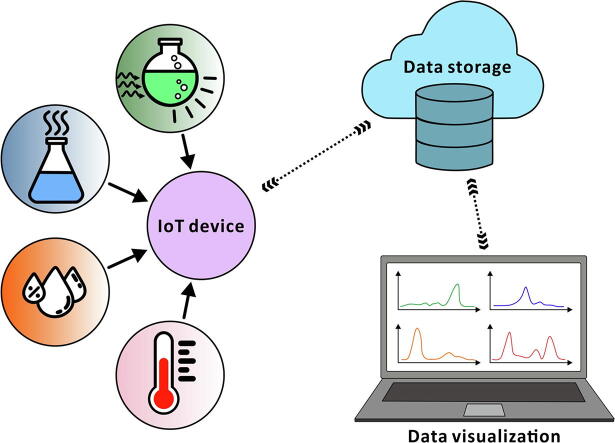

Specifications table

**Table:** 

Hardware name	Telechemistry 2.0
Subject area	● Chemistry ● General
Hardware type	● Field measurements and sensors ● Measuring physical properties and in-lab sensors
Open Source License	MIT license
Cost of Hardware	$ 293.73 USD
Source File Repository	https://doi.org/10.17605/OSF.IO/7PHR5

## 1 Hardware in context

In the past decade, a number of chemists have dedicated themselves to integrate modern electronics into conventional chemical laboratories to build customized experimental systems [1]. Microcontroller boards (MCBs) and single-board computers (SBCs) have been widely implemented due to their advantages, such as small form factor, low cost, open-source design, and flexibility to integrate with laboratory equipment [2], [3], [4], [5], [6]. The accessibility of open-source hardware and software further increases the possibility of developing new instruments [7]. Moreover, with the recent advances in three-dimensional (3D) printing technology, it is possible to design and construct devices to meet experimental needs [8], [9]. By utilizing these modern tools, one can automate trivial experimental and data handling steps, and achieve a high working efficiency. Furthermore, the Internet-of-Things (IoT) has emerged as a promising tool not only to the general public but also to chemists [10]. The ability to create a network of electrical appliances, sensors, cloud storage facilities, and devices, enables exchange of information between the components in the network and the researchers [11]. The advantages of such a system include: remote access to experimental setups and data, ability to share data among researchers by surpassing the limitations of space and time, transparency in experimental data handling, minimizing human exposure to hazardous chemical environments, to name a few. Several studies have demonstrated the usefulness of the technology in facilitating laboratory activities, for example, remote monitoring of (bio)chemical processes and laboratory environment [12], monitoring chemical reaction with a smart stirrer [13], tracking sample vials [14], and monitoring instrument performance [15]. This study takes advantage of off-the-shelf electronic components and 3D-printing technology to build a cloud-integrated system for real-time monitoring of fluorescent chemical reactions and the laboratory environmental parameters (*e.g.* temperature, humidity, concentration of flammable vapors).

Fluorescence measurements are routinely performed in chemical laboratories that specialize in synthesis and study of fluorescent chemicals. High-end commercial fluorescence spectrometers have been the first choice for such measurements. However, the current open-source hardware movement as well as the availability of inexpensive electronic and optical components have enabled chemists to build customized fluorescence detection systems. For example, Szymula *et al.* developed an open-source plate reader that can be used for the measurement of fluorescence as well as absorbance/optical density of several samples on a standard multiwell plate [16]. A previous study from our lab also took advantage of electronic and optical tools, and 3D-printed components to measure the fluorescence of an acoustically actuated microliter sized droplet pinned on a hydrophobic thread [17]. While these setups reduce the sample volume required for fluorescence measurement, there also exist fluorometric probes developed by the medical research community for *in vivo* medical diagnosis (*e.g.* endoscopy) [18], [19], [20], [21]. In the present study, we use a similar concept to design a probe for *in situ* fluorescence measurement in a chemical reactor for real-time and remote monitoring of a long-term reaction that yields a fluorescent product.

## 2 Hardware description

In a conventional fluorescence spectrometer, about a few milliliters of sample are placed in a cuvette, the sample is excited with light at an appropriate wavelength, and the light emitted at another wavelength is measured. On the other hand, a microtiter plate-based fluorescence reader requires a few microliters of the sample. In either case, one would have to transfer some volume of sample from a reaction vessel to the sample holder for fluorescence measurement. Alternatively, one could measure the fluorescence using an automated continuous-flow platform, in which the sample is continuously passed through a custom designed flow cell with orthogonally fitted optical fibers for the transmission of excitation and emitted lights [22]. The automated setup uses light from a light-emitting diode (LED) for excitation, and a commercial spectrophotometer as a detection unit for emitted light [22].

**Fig. 1:**
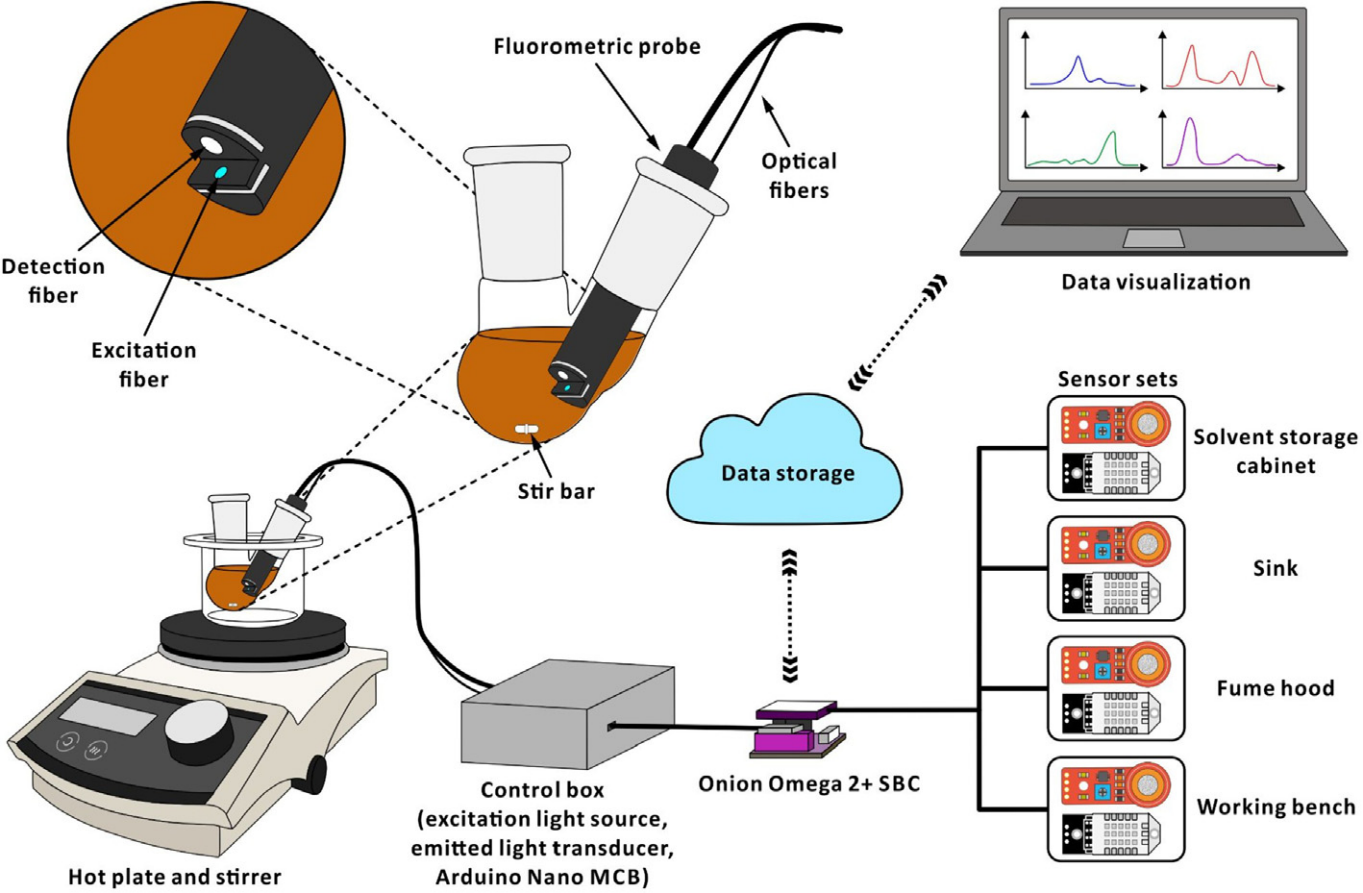
Schematic diagram showing the Telechemistry 2.0 setup.

The fluorometric probe—described in this study—incorporates an in-house-built 3D-printed probe, two optical fibers, an LED, a photoresistor, and an MCB (Fig. 1). The probe is designed to fit into batch type reactors (*e.g.* multi-neck spherical flask) for monitoring long-term reactions. The probe is directly dipped into the reaction mixture, and two optical fibers drawn from the probe facilitate the transmission of the excitation and emitted light. While the LED—installed at the other end of one optical fiber—is an excitation light source, the photoresistor—fitted at the other end of the second optical fiber—detects the emitted light from the reaction mixture. The modular design of the device allows one to easily replace the LED or the photoresistor with those that are suitable for other excitation and emission wavelengths. The associated MCB collects the data acquired from the photoresistor, and transmits these data to an SBC. The data from the MCB and the auxiliary sensors (temperature, humidity, and alcohol vapor) are uploaded to a cloud storage platform by the SBC. The advantages of the system include:

● The device can be easily built using inexpensive off-the-shelf electronic components, optical fibers, and 3D-printed components.
● Computers or hand-held devices with network connectivity can be used to remotely visualize the experimental data.
● The device has a modular design, is easy to operate, and can be directly fitted to a conventional reaction flask.
● Inexpensive sensors facilitate monitoring laboratory environmental parameters for safety purpose.

## 3 Design files

### 3.1 Electronics

The setup takes advantage of a low-cost SBC (Onion Omega 2+ [23]) to collect data from the sensors. The SBC with a small form factor (34 mm × 20 mm) features a microprocessor (chipset, MT7688 SoC; architecture, MIPS24KEc) operating at a clock speed of 580 MHz with an associated random access memory (RAM) of 128 MB. The module has an on-board flash memory of 32 MB and supports Internet connectivity (2.4 GHz 802.11b/g/n Wi-Fi). It runs on a Linux operating system (based on OpenWrt Project [24]), and can be programmed in popular programming languages such as C, C++, and Python. Moreover, the module incorporates standard interfaces, such as 18 general purpose input/output (GPIO) pins, TX/RX pins, and a set of I^2^C pins, which enable communication with auxiliary components (*e.g.* sensors or other electronic controllers). In our setup, we use TX/RX pins to enable serial communication between the SBC and an MCB (Arduino Nano [25]) which is used to collect the fluorescence data. Arduino Nano comprises a microcontroller (chipset, ATmega328; architecture, AVR) with a processing power of 16 MHz, a flash memory of 32 kB, and 2 kB RAM, on an 18 mm × 45 mm printed circuit board (PCB). In addition, an analog-to-digital converter (ADC, 16-bit resolution) expansion board [26] is attached to the SBC to convert the analog signals from the auxiliary sensors to digital values. Assigning a separate MCB for fluorescence data collection enables dedicating the MCB’s processing power on a single task, while the SBC uses its processing power on multiple tasks such as data collection from auxiliary sensors, Wi-Fi connectivity, and data transmission to the cloud.

**Fig. 2:**
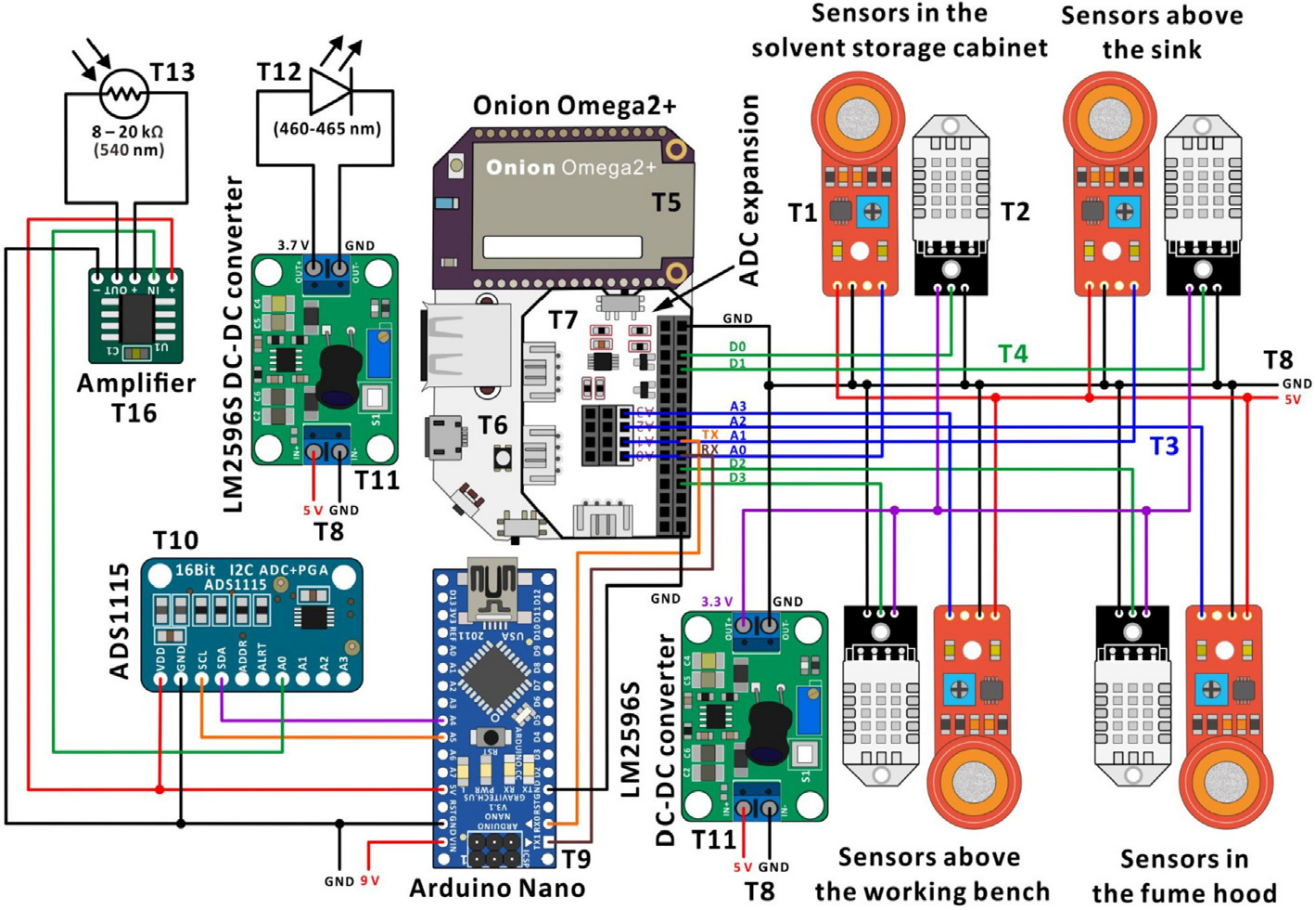
The electronic circuit used in the Telechemistry 2.0 device.

The intensity of fluorescence light detected using a photoresistor (resistance range: 8–20 kΩ, wavelength range: 350–780 nm, highest sensitivity at: 540 nm) is recorded by the MCB *via* an amplifier and an external ADC (Fig. 2). The photoresistor is connected to a custom-made amplifier [27] PCB, incorporating an OP07 operational amplifier to amplify the signals. The output of the amplifier PCB is connected to an external ADC (ADS1115, 16-bit resolution) set up for the MCB. The data is transmitted from the ADC to the MCB, and is further uploaded to the cloud by the SBC. A blue LED of emission wavelength 460–465 nm (power = 3 W) is used as an excitation light source. The LED is soldered onto a heatsink to dissipate the excess heat generated during its operation, and is powered through a DC-DC converter (LM2596S) connected to a wall socket using an AC-DC adapter (input, 110 V; output, 5 V; current, 2 A). The input voltage for the DC-DC converter is 5.0 V while the output voltage is set to 3.7 V, to provide a stable power to the LED. The TX/RX pins are used to establish a serial communication between the MCB and the SBC.

To demonstrate the feasibility of the setup in monitoring laboratory environmental conditions, the Telechemistry 2.0 setup also incorporates four sets of sensors. Each set contains an alcohol vapor sensor (MQ3) and a temperature cum humidity sensor (DHT22). We choose MQ3 and DHT22 sensors because ethanol is regularly used in our laboratory, and ambient temperature as well as humidity often affect the outcomes of the experiments. Moreover, MQ3 and DHT22 sensors are inexpensive and are readily available in local electronic shops. One could choose different sensors (see for example [28]) depending on what one wants to monitor in the laboratory. The four MQ3 sensors and DHT22 sensors used in our setup are connected to the ADC expansion board of the SBC, and are powered through a wall socket using an AC-DC adapter (input, 110 V; output, 5 V; current, 2 A). The DHT22 sensors are connected to a DC-DC converter (LM2596S) that converts 5 V to 3.3 V to provide stable power to the sensors.

### 3.2 Mechanical design

**Fig. 3:**
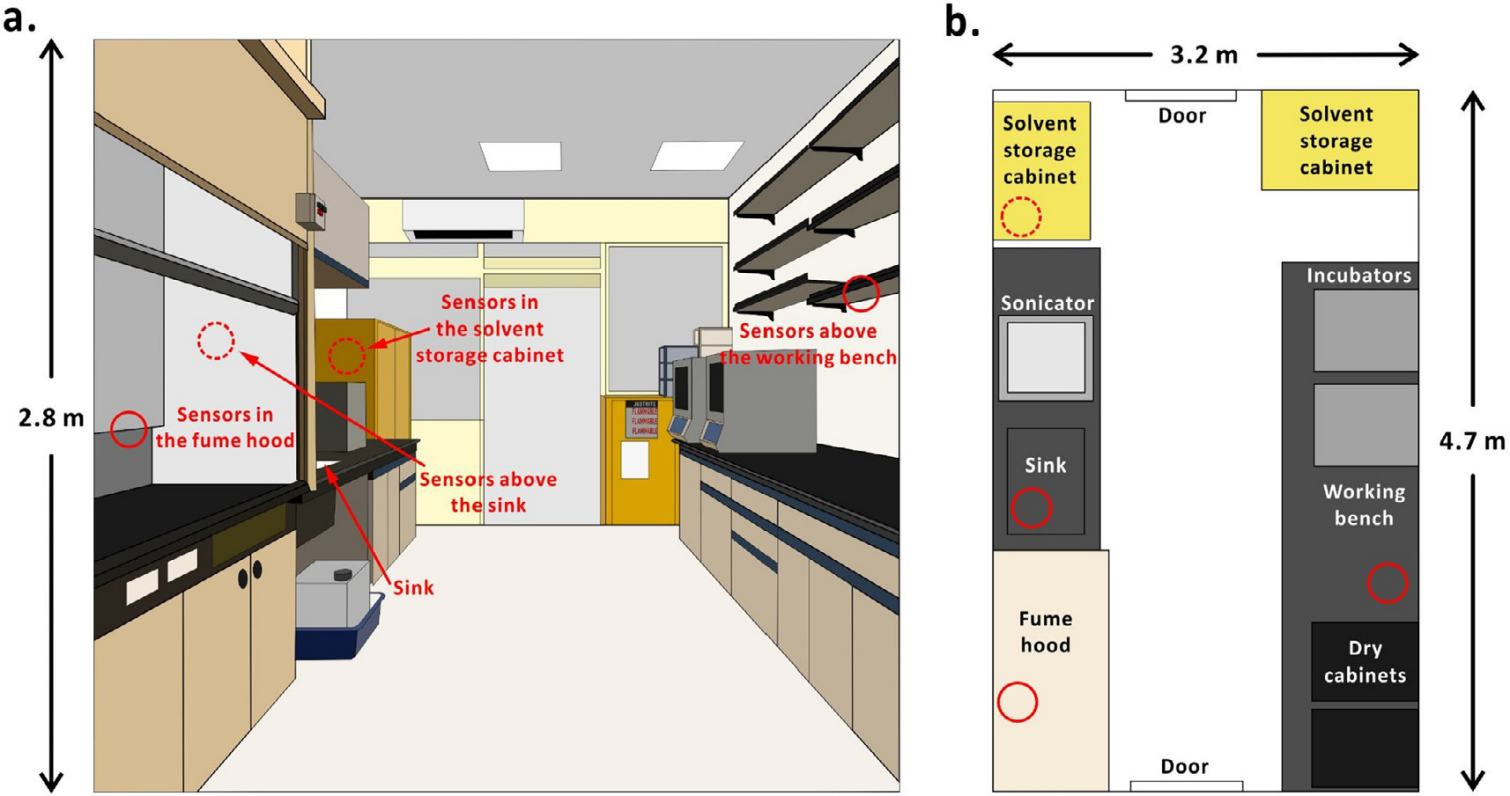
Sensor positions in a chemical laboratory. (a) Side view of the location of sensors; (b) top view of the location of sensors. Red circles represent the sensor positions. (For interpretation of the references to colour in this figure legend, the reader is referred to the web version of this article.)

The MQ3 and DHT22 sensors are fixed in four different locations in a chemical laboratory: inside a storage cabinet for ethanol, above a sink, in a fume hood, and above a working bench (Fig. 3). These are the places where ethanol is often utilized or stored. The core of the device is the SBC that is fixed in a box and put on a shelf above the sink. MQ3 sensors are connected using long flat rainbow ribbon cables and DHT22 sensors are connected using 20-AWG cables (3.0 m each for sensors inside the solvent storage cabinet and for the ones in the fume hood, 0.5 m for the ones above the sink, 7.5 m for the ones above the working bench), and are fixed onto the wall with insulation tape.

**Fig. 4:**
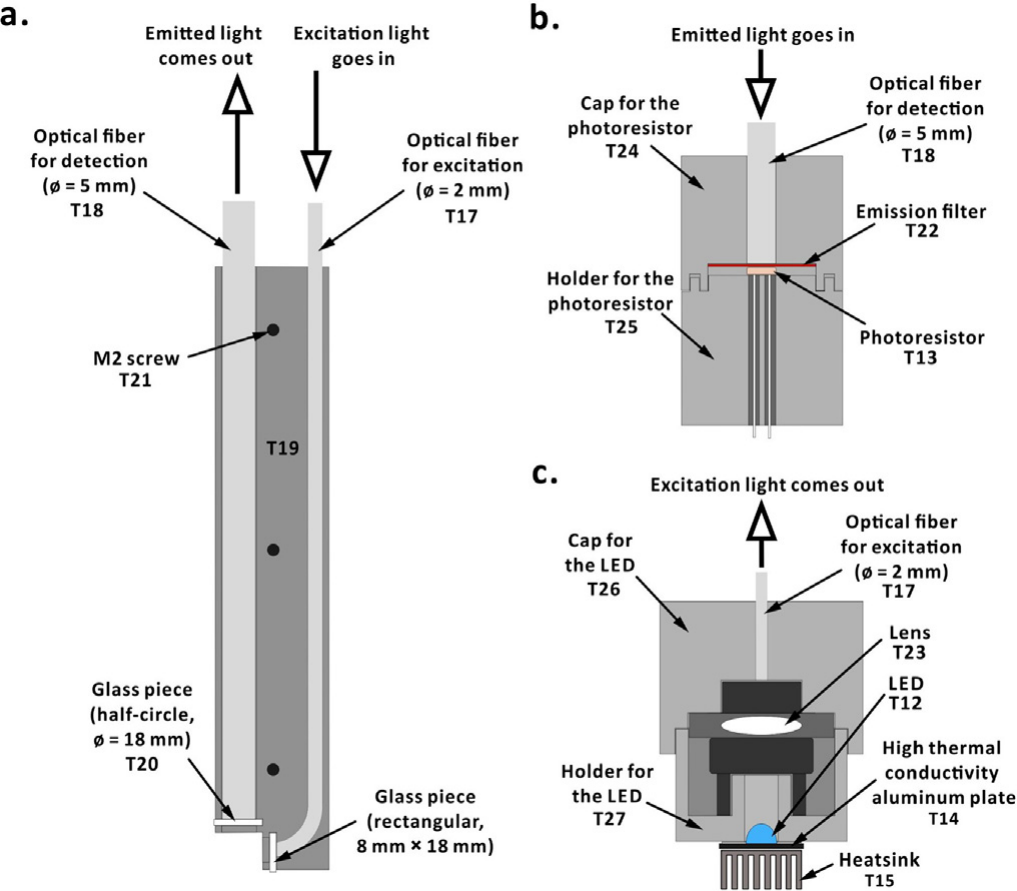
Designs of the 3D-printed components. (a) Cross sectional views of the fluorescent probe; (b) photoresistor holder; (c) LED holder.

**Fig. 5:**
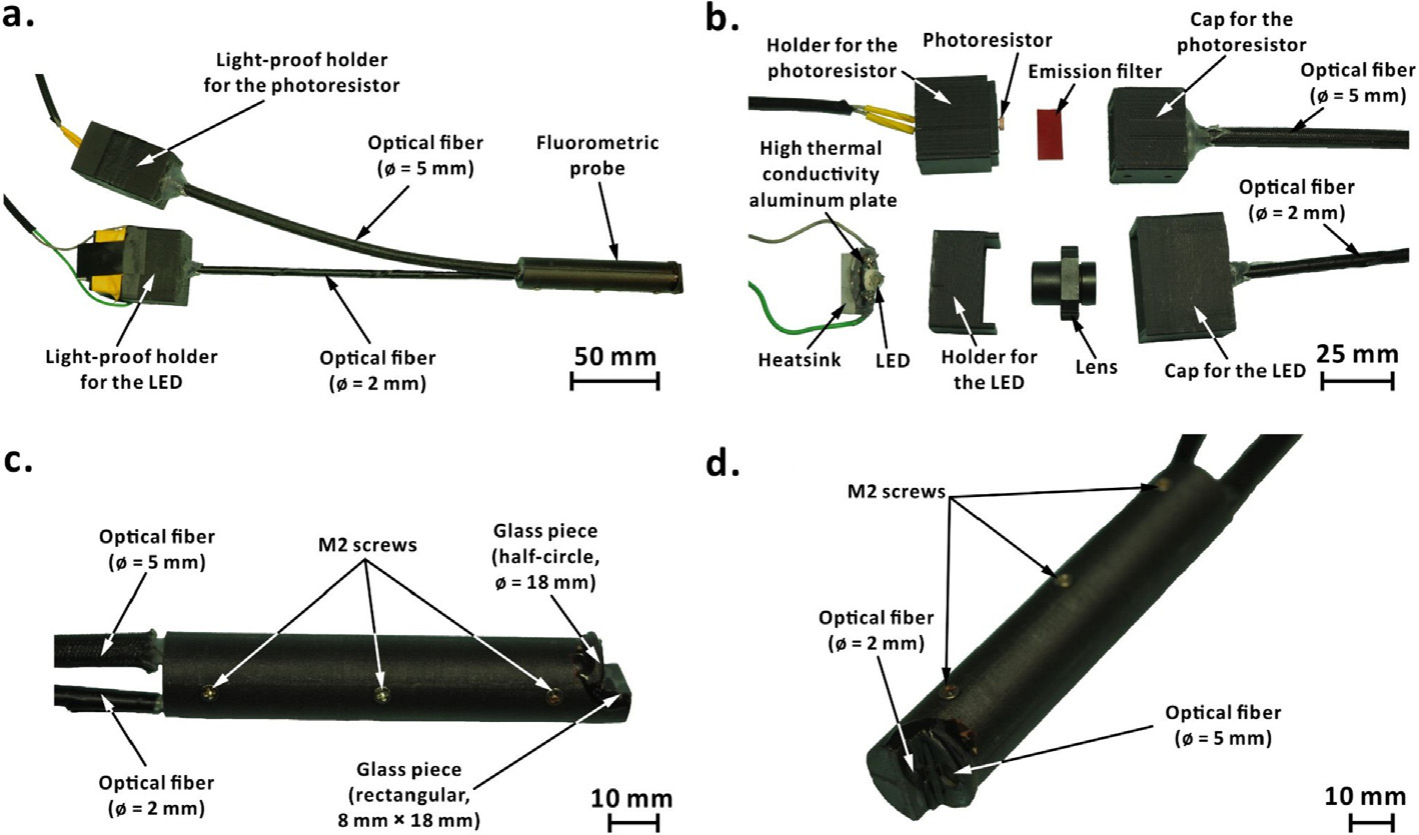
Photographs of the fluorescence detection system used in Telechemistry 2.0 setup. (a) 3D-printed fluorometric probe with optical fibers and 3D-printed light-proof holders for LED and photoresistor; (b) components inside the disassembled light-proof holders; (c) side view of the fluorometric probe; (d) photograph depicting the orthogonal alignment of optical fibers at the fluorometric probe-head.

The fluorometric probe in the Telechemistry 2.0 device is 3D-printed in two halves using polyetheretherketone (PEEK; *cf.* Section 4) as substrate. Channels are designed in the two halves of the probe to hold two optical fibers in place (Fig. 4, Fig. 5). One optical fiber [ø = 2 mm; length = 200 mm; core material, poly(methyl methacrylate); jacket material, polytetrafluoroethylene] is for transmitting the excitation light from the LED to the reaction mixture. Another optical fiber [ø = 5 mm; length = 200 mm; core material, poly(methyl methacrylate); jacket material, polytetrafluoroethylene] is to transmit emitted light from the reaction mixture to the photoresistor. The orthogonal alignment of optical fibers at the probe-head reduces possible interference of excitation light with emitted light. As the probe-head will be in contact with the reaction mixture, two 1-mm thick glass pieces are placed and glued (material, epoxy resin) in the front of the optical fibers to protect the optical fibers from chemicals. A glass piece of rectangular shape (8 mm × 18 mm) is placed in front of the excitation fiber and another glass piece of a half-circle shape (ø = 18 mm) is placed in front of the emission fiber. The other end of the emission fiber is inserted into a light-proof holder for a photoresistor. The light-proof holder consists of two 3D-printed parts and an emission filter [red light filter with transmission wavelength 580–700 nm (026, Rosco, Stamford, CT, USA; maximum transmittance wavelength, 740 nm) or green light filter with transmission wavelength 420–580 nm (090, Rosco, Stamford, CT, USA; maximum transmittance wavelength, 520 nm)] to filter unwanted wavelengths of light. It is possible to use multiple layers of filters as demonstrated below (*cf.* Section 7). Likewise, the other end of the excitation fiber is inserted into another light-proof holder for an LED. The LED holder consists of two 3D-printed parts and a lens (ø = 6 mm; thickness, 2 mm; focal length, 10 mm ± 1 mm) to focus the excitation light into the optical fiber. In addition, the LED is soldered onto a high thermal conductivity plate (20.9 mm × 19.9 mm; material, aluminum) with a heatsink (20 mm × 14 mm × 6 mm; material, aluminum). All the 3D-printed parts were designed using Inventor Professional 2020 software (version 2020.0.1; Autodesk, San Rafael, CA, USA).

### 3.3 Software

The SBC can be accessed from a personal computer (PC) through a free and open-source terminal emulator cum serial console called PuTTY [29]. Besides, wireless connection between the SBC and any PC can be established through the web-based Onion Console [30]. Note that the PC and the SBC should be connected to the same Wi-Fi network. In the present study, the programming language used to control the SBC is Python. The program includes instructions for the SBC to acquire fluorescence data from the MCB, analog signals from the MQ3 sensors and digital data from the DHT22 sensors. Furthermore, using an inputted calibration equation, the program calculates the concentration of alcohol vapour and finally transfers all the data (at a rate of ∼ 0.004 Hz for MQ3 sensors and DHT22 sensors; at a rate of ∼ 0.03 Hz for fluorescence data) to the cloud storage platform called *ThingSpeak* [31]. The separate channels set up on the *ThingSpeak* platform for data from different sensors enable remote monitoring of the reaction progress and the laboratory environment conditions. To program the MCB, a free and open-source software Arduino Integrated Development Environment (IDE) [32] can be used.

### 3.4 Design files summary

**Table:** 

Design file name	File type	Open source license	Location of the file
Electronic circuit	figure	MIT license	available at: https://doi.org/10.17605/OSF.IO/7PHR5
3D design file	.stl		
Onion_Omega	.py		
Arduino_Nano	.ino		
Fluorometric device	.mp4		

*Electronic circuit*: An illustrative electronic circuit diagram of the setup.

*3D design file*: Design file used for 3D-printing the fluorescence detection probe.

*Onion_Omega*: Python source code for SBC. The program consists of instructions for the SBC to collect data from sensors and upload them to the cloud.

*Arduino_Nano*: Arduino code for MCB. The code consists of instructions for the MCB to collect data from the photoresistor and transmit it to the SBC.

*Fluorometric device*: A video of assembling the fluorometric device. The video consists of three parts for assembling the light-proof holder for the photoresistor, the light-proof holder for the LED, and the fluorometric probe.

## 4 Bill of Materials

**Table:** 

Label in Fig. 2, Fig. 4	Item	Quantity	Cost per unit (USD)	Total cost (USD)	Supplier	Catalog number	Material type
T1	MQ3 (alcohol sensor)	4	3.16	12.64	Kinsten, https://kinsten.com.tw/	2D0075	Electronics
T2	DHT22 (temperature cum humidity sensor)	4	6.40	25.60		2D060	Electronics
T3	Flat rainbow ribbon cables (14 m)	1	9.50	9.50		WAD0018	Electronics
T4	20-AWG cable (14 m)	1	9.51	9.51		WAK0082	Electronics
T5	Onion Omega 2 + SBC	1	15.35	15.35	Onion Store, https://onion.io/store/	OM-O2P	Electronics
T6	Onion Omega expansion dock	1	15.00	15.00		OM-D-EXP	Electronics
T7	Onion Omega ADC expansion board	1	15.00	15.00		OM-E-ADC	Electronics
T8	Standard type A to micro B USB 2.0 cable	3	1.76	5.28	Centenary Materials, http://us.100y.com.tw/	107,551	Electronics
T9	Arduino Nano MCB	1	20.70	20.70		98,443	Electronics
T10	ADS_1115 ADC	1	4.43	4.43		136,511	Electronics
T11	LM2596S DC-DC converter	2	3.51	7.02		96,038	Electronics
T12	LED (460–465 nm)	1	0.88	0.88		96,662	Electronics
T13	Photoresistor (8–20 kΩ, 350–780 nm, highest sensitivity: 540 nm)	1	0.13	0.13		53,901	Electronics
T14	High thermal conductivity aluminum plate	1	0.07	0.07		91,069	Electronics
T15	Heatsink	1	1.25	1.25		138,583	Electronics
T16	Custom-made amplifier PCB (*cf.*[43])	1	13.28	13.28	Good Technology, Taichung, Taiwan	–	Electronics
T17	Optical fiber (Ø = 2 mm)	1	5.55	5.55	Shiner Fiber Optics, Hsinchu, Taiwan	TW-0200	Optics
T18	Optical fiber (Ø = 5 mm)	1	11.07	11.07		TW-0500	Optics
T19	3D printed probe	1	40.62	40.62	Brusat, Taichung, Taiwan	–	3D-printing (PEEK)
T20	Glass piece	2	5.27	10.54	NTHU workshop, Hsinchu, Taiwan	–	Optics
T21	M2 screw	3	0.07	0.21	Kinsten, https://kinsten.com.tw/	AWA002-008	Electronics
T22	Emission filter	1	27.73	27.73	Rosco, Stamford, CT, USA	100 0R26S 2024	Optics
T23	Lens	1	7.94	7.94	Koodyz Technology, Taipei County, Taiwan	–	Optics
T24, T25, T26, T27	light-proof holders and caps	4	–	–	–	–	3D-printing(ABS)
	Perfboard	2	0.35	0.70	Centenary Materials, http://us.100y.com.tw/	2826	Electronics
	Insulation tape	1	0.63	0.63		6640	Electronics
	Thermal grease	1	1.50	1.50		101,464	Polymer
	AC to DC wall adaptor (AC 110 V to DC 9 V)	1	7.08	7.08		104,583	Electronics
	AC to DC wall adaptor (AC 110 V to DC 5 V)	4	5.33	21.32		140,439	Electronics
	Dupont wires	36	0.05	1.80	Kinsten, https://kinsten.com.tw/	CBC001	Electronics
	Epoxy glue	1	1.40	1.40	Local hardware store, Hsinchu, Taiwan	CK-080	Polymer

### 4.1 Chemicals

Fluorescein, Bovine Serum Albumin (BSA) (98+%, heat shock fraction, pH = 7), lipase (from *Candida antarctica*, specific activity: ≥5,000 U g^−1^, immobilized on acrylic resin) and propyl butyrate (99 %) were from Sigma-Aldrich (St. Louis, MO, USA). Ammonium acetate (98+% for HPLC) was from Acros Organics (Geel, Belgium). Deionized water, Sodium hydroxide (99+%) and hydrochloric acid were from Merck (Darmstadt, Germany). Hydrogen tetrachloroaurate(III) tetrahydrate was from Showa Chemical Industry (Tokyo, Japan). Ethanol (99.5+%) was from Echo Chemical (Miaoli, Taiwan).

## 5 Build instructions

### 5.1 Setting up the Onion Omega 2 + SBC

To begin with, mount the SBC onto the expansion dock, and connect the expansion dock to a PC with a standard type A–micro B USB 2.0 cable to power up the SBC as well as to establish a serial connection with the PC. To boot up the SBC, turn on the power switch of the expansion dock. The amber LED on the SBC will blink, indicating the system is booting. After finishing the boot sequence, the amber LED will stop blinking and stay bright. In this case, one can start to control and program the SBC through the PC. To know the serial port of connection for the SBC, go to the ‘Device manager’ of the PC and check under ‘Ports (COM & LPT)’. Note down the COM port number (*e.g.* COM1). On the PC, download and install PuTTY [29]. Launch the PuTTY application, and select ‘Serial’ for the connection type. Then, enter the previously noted COM port number in the ‘Serial line’ entry bar and enter ‘115200′ in the ‘Speed’ entry bar, and click ‘Open’ to open the terminal.

#### 5.1.1 Connecting the SBC to a Wi-Fi network

Run the command ‘wifisetup’ in the command line of the PuTTY terminal. There will be three options shown, select the first one, ‘scan for Wi-Fi networks’ by entering ‘1′. The scanning will take a few seconds to search for available networks. After that, enter the number corresponding to the Wi-Fi network. Lastly, enter the password of the chosen Wi-Fi network, if any. The network adapter restarts and completes the connection to the chosen Wi-Fi network in about 30 s. It is not necessary to repeat this step every time while powering up the SBC, unless one wants to connect to another Wi-Fi network. The SBC can automatically connect to the chosen Wi-Fi network while booting.

#### 5.1.2 Installing the software on the SBC

After connecting the SBC to a Wi-Fi network, one can start installing the software necessary to operate the device. First, download and install Python3 on the SBC by running the commands ‘opkg update’ and ‘opkg install python3′, one after the other. It takes about one minute to complete the installation. Then, install the official Python package manager (Python Install Program (pip)) by running the command ‘opkg install python3-pip’. Followed by, upgrade the setuptools package by running the command ‘pip3 install --upgrade setuptools’, otherwise, the following Python packages would fail to install. Install the packages by running the commands ‘pip3 install paho_mqtt’, ‘pip3 install pyserial’, and then install the library for DHT22 sensor by running ‘opkg install dht-sensor’. Lastly, run the following commands one by one to install the packages for the MQ3 sensor: ‘opkg install python3-adc-exp’, ‘opkg install pyOnionI2C’, and ‘opkg install pyOmegaExpansion’.

On the PC, download the ‘Onion_Omega.py’ file (*cf*. Section 3.4). One could use the Python IDLE [33] on the PC to take a look or edit the Python code. In the SBC, create a similar file by running ‘vi Onion_Omega.py’ in the PuTTY command line to open the Vim text editor page. Press ‘i’ to open the editing mode before editing/inserting script to the file. After editing/inserting the script, press ‘Esc’ to save the file and enter the command mode. In the command mode, enter ‘:wq’ to exit the Vim text editor, and the ‘Onion_Omega.py’ file will be ready.

### 5.2 Setting up the Arduino Nano MCB

On the PC, download and install the Arduino IDE software [32]. Disconnect the SBC and connect the MCB to the PC with a standard type A–micro B USB 2.0 cable. To know the serial port of connection for the MCB, open the ‘Device manager’ on the PC and check under ‘Ports (COM & LPT)’. Note down the COM port number (*e.g.* COM1). In the Arduino IDE, click ‘Tools’ on the menu bar, a dropdown list will appear. Hover the mouse over the ‘Board’ button and choose ‘Arduino Nano’ visible under the ‘Arduino AVR Boards’. Then, hover the mouse over the ‘Port’ button just below the ‘Board’ button, and choose the COM port of Arduino Nano. Download the ‘Arduino_Nano.ino’ file from the source file repository (*cf*. Section 3.4). Then, upload the code to the MCB by clicking the upload button (a round shaped button with a right arrow symbol) on the Arduino IDE. After that, disconnect the MCB from the PC, and put the MCB in the control box of Telechemistry 2.0 device and connect to the SBC to establish serial connection *via* TX/RX pins.

### 5.3 Setting up the fluorometric device

Download the 3D design files from the source file repository (*cf*. Section 3.4) and print the components of the light-proof holders for photoresistor and LED, using a 3D-printer. In our case, we used a fused-deposition-modeling 3D-printer (UP Plus, 3DP-14-4D; Beijing TierTime Technology, Beijing, China) with acrylonitrile–butadienestyrene (ABS) as printing material. Once the 3D printed components are ready, fit the photoresistor inside its holder (T25 in Fig. 4; see the supporting video file ‘Fluorometric device.mp4′ in the source file repository) and place the emission filter inside the cap (T24 in Fig. 4). Put together the holder and the cap to form a light-proof holder for the photoresistor. Insert the optical fiber (Ø = 5 mm) through the cap’s hole, such that the open end of the fiber touches the emission filter (Fig. 4**b**). Use thermal glue or epoxy resin to firmly fix the optical fiber to the cap. Similarly, fit the lens between two parts (T26 and T27 in Fig. 4) of the LED’s light-proof holder, and insert the optical fiber (Ø = 2 mm) through its cap’s hole. Solder the LED onto the high thermal conductivity aluminum plate and apply a layer of thermal grease on the backside of the aluminum plate. Adhere the heatsink on the thermal grease and fit the LED with heatsink to the light-proof holder using insulation tape. Then, fix the two parts of the fluorometric probe with three M2 screws and insert the other ends of the two optical fibers into the fluorometric probe. Place the two glass pieces and glue them with epoxy resin. Connect the photoresistor and the LED to the rest of the circuit using dupont wires according to the electronic scheme shown in Fig. 2.

### 5.4 Setting up the environmental sensors

To set up the MQ3 and DHT22 sensors, prepare the wires of appropriate lengths and make the connections as shown in the electronic scheme in Fig. 2. Note that there will be a drop in voltage with longer cable lengths, and this can disrupt the signals from the sensors. Stick the wires along the walls using insulation tapes for safety purposes. Attach the sensors to a solid support (wall or ceiling) using insulation tapes.

### 5.5 Setting up the IoT platform

Create an account on the ThingSpeak IoT platform [31] and follow the instructions in this MathWorks tutorial [34] to set up the channels for the deposition of experimental data. Briefly, create four new channels in the account to deposit the data from sensors, one each for alcohol, temperature, humidity and fluorescence measurements. In the channels for alcohol, temperature and humidity data, visit the “Channel Settings” tab of each channel one-by-one and tick the checkboxes for four fields. These four fields will be used for recording the data from the sensors placed at four different positions in the laboratory. For example, in our case, field 1 corresponds to the sensors inside the solvent storage cabinet, field 2 corresponds to the sensors above the sink, field 3 corresponds to the sensors inside the fume hood, and field 4 corresponds to the sensors above the working bench. Name the fields as per the sensors’ positions and click ‘Save Channel’ at the bottom of the webpage. Then, move to the ‘API Keys’ tab and copy the ‘Write API’ code and the ‘channel ID’ number from every channel. Lastly, insert the API keys and channel IDs into the ‘Onion_Omega.py’ script to specify the channels and fields to the SBC for the deposition of data from sensors to the IoT platform. Please see the instructive statements included in the supporting file ‘Onion_Omega.py’, to know where to paste these channel and field credentials in the script.

## 6 Operation instructions

1. Plug the four AC-DC adapters (input: 110 V; output: 5 V; current: 2 A) to the wall sockets to power up the SBC, MQ3 sensors, DHT22 sensors and the LED.
2. Similarly, plug the AC-DC wall adaptor (input: 110 V; output; 9 V; current: 1 A) to a wall socket for powering up the MCB.
3. To run the Python code on the SBC, first, open the web-based Onion Console on a PC by entering ‘http://omega-abcd.local/’ in its Internet browser (*e.g.* Google Chrome). Note that the PC should be connected to the same Wi-Fi network as the SBC. In the Onion Console, the “abcd” represents the ID mentioned on the SBC. In the command line, enter ‘python3 Onion_Omega.py’ to start the program. Then, check the ThingSpeak channels to know if the sensor data is being uploaded to the IoT platform.
4. Set up the reaction in a fume hood for safety reasons. While performing experiments, immerse the fluorometric probe-head into the reaction mixture and use a three-prong clamp to fix the probe to a stand (Fig. 6). The PEEK material—used to print the probe—is compatible with common organic solvents, is resistant to acid and base solutions, and is stable at high temperatures [35], [36]. Therefore, the probe is suitable for a number of fluorescent chemical reactions.

**Fig. 6:**
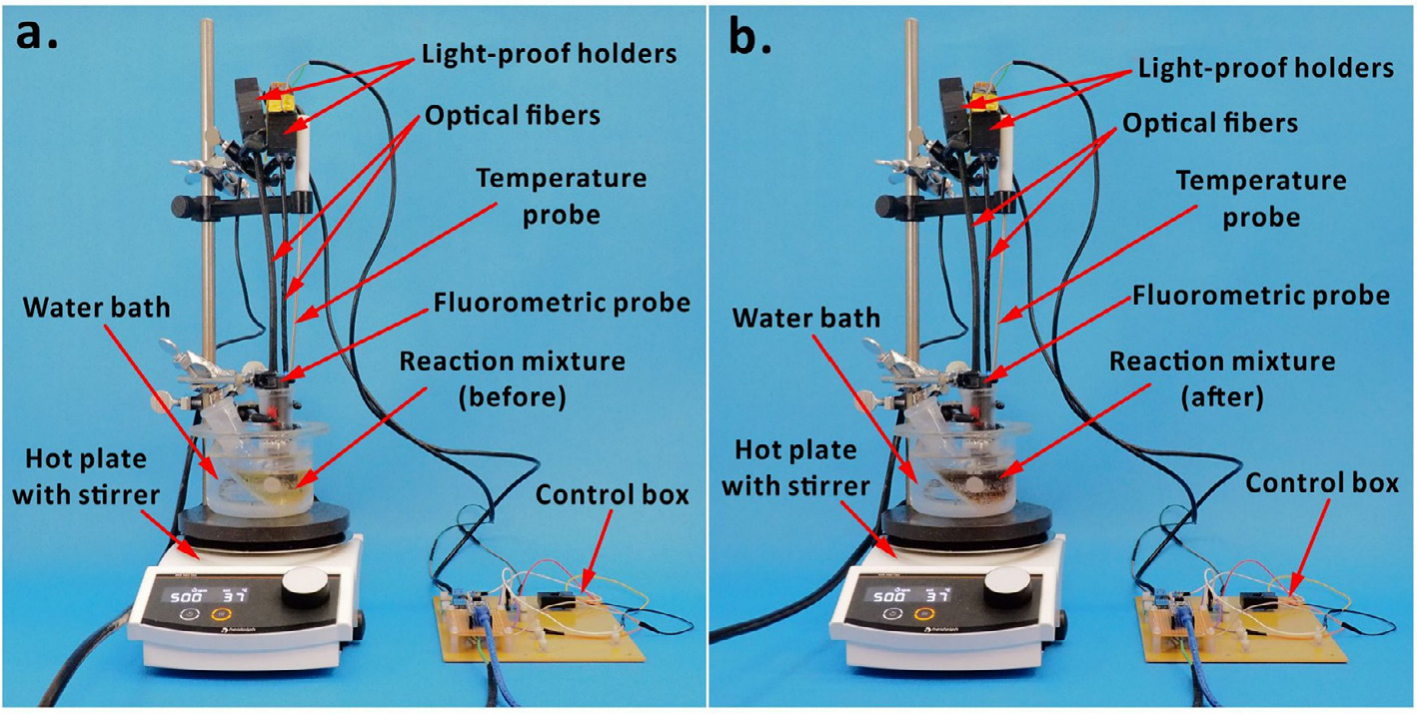
Photographs of reaction setup. (a) Before the reaction and (b) after the reaction.
5. After use, carefully wash the probe with deionized water and wipe using tissue paper. If the probe comes in contact with organic solvents, use ethanol to wash it.

## 7 Validation and characterization

### 7.1 Calibration of the sensors

**Fig. 7:**
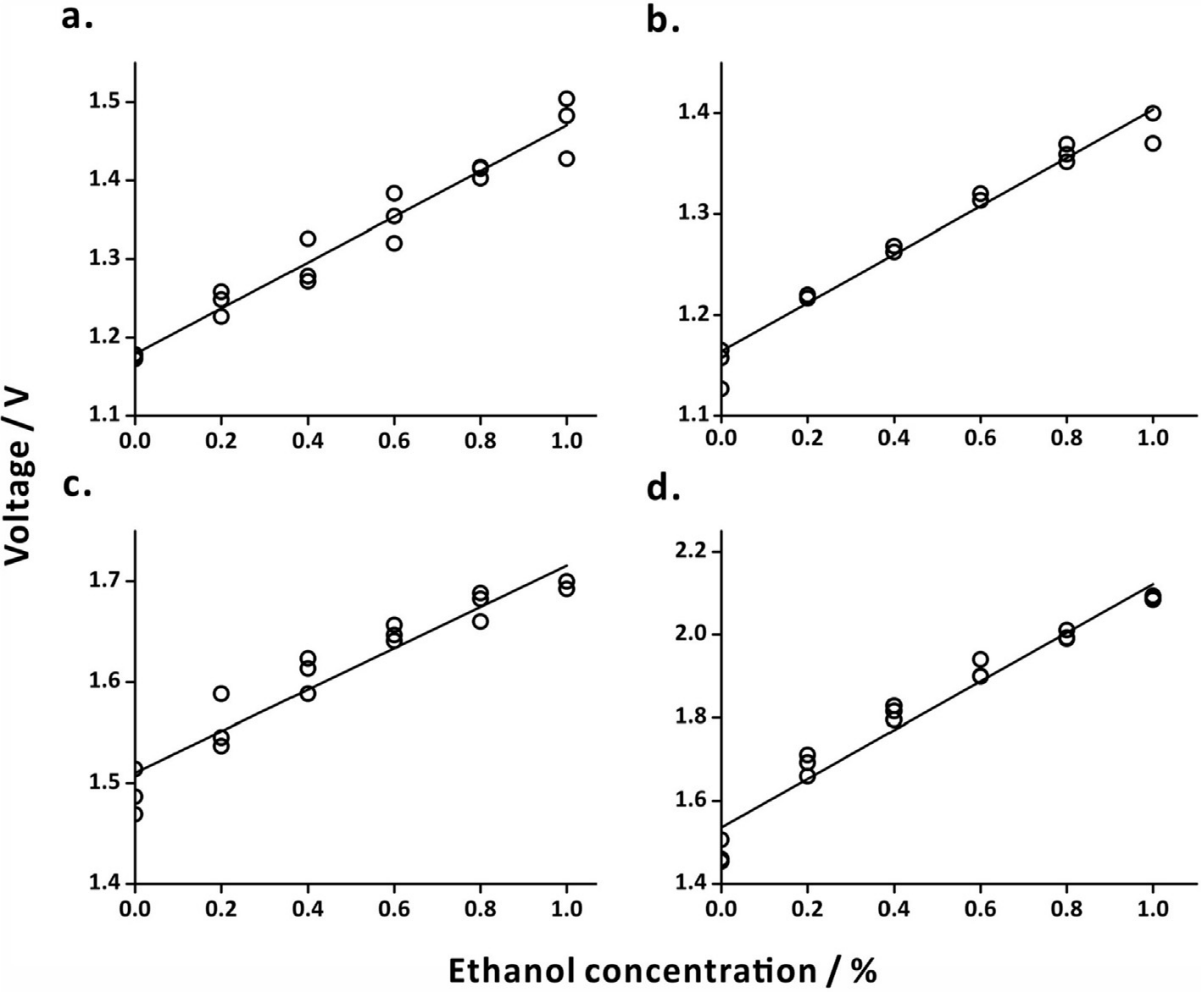
Calibration of alcohol sensors setup at different locations in the laboratory. (a) In the solvent storage cabinet, calibration equation: *Voltage* = (0.29 ± 0.02) × *Ethanol concentration* + (1.18 ± 0.01), *R*^2^ = 0.96; (b) above the sink, calibration equation: *Voltage* = (0.24 ± 0.01) × *Ethanol concentration* + (1.16 ± 0.01), *R*^2^ = 0.97; (c) in the fume hood, calibration equation: *Voltage* = (0.21 ± 0.01) × *Ethanol concentration* + (1.51 ± 0.01), *R*^2^ = 0.92; (d) above the working bench, calibration equation: *Voltage* = (0.59 ± 0.03) × *Ethanol concentration* + (1.54 ± 0.02), *R*^2^ = 0.96. In all cases, *n* = 3.

The MQ3 sensors were calibrated using ethanol solutions of concentrations 0.0, 0.2, 0.4, 0.6, 0.8, and 1.0% (v/v) in water in 50-mL vials. During calibration, the sensors were fixed through holes drilled on the caps of the vials in such a way that the sensing zone was facing the inside of the vial. Thermal glue was used to fill any gaps between the sensor and the cap to ensure the system remained closed during calibration. The voltage signals of the four MQ3 sensors showed linear trends corresponding to the different concentrations of ethanol solutions (Fig. 7). During calibration, the lengths of the wires were kept the same as in the final setup, this was to make sure that there were no voltage drops from the MQ3 sensors due to the length of the wires. Note that it is not necessary to calibrate the DHT22 sensors because they are factory-calibrated sensors.

**Fig. 8:**
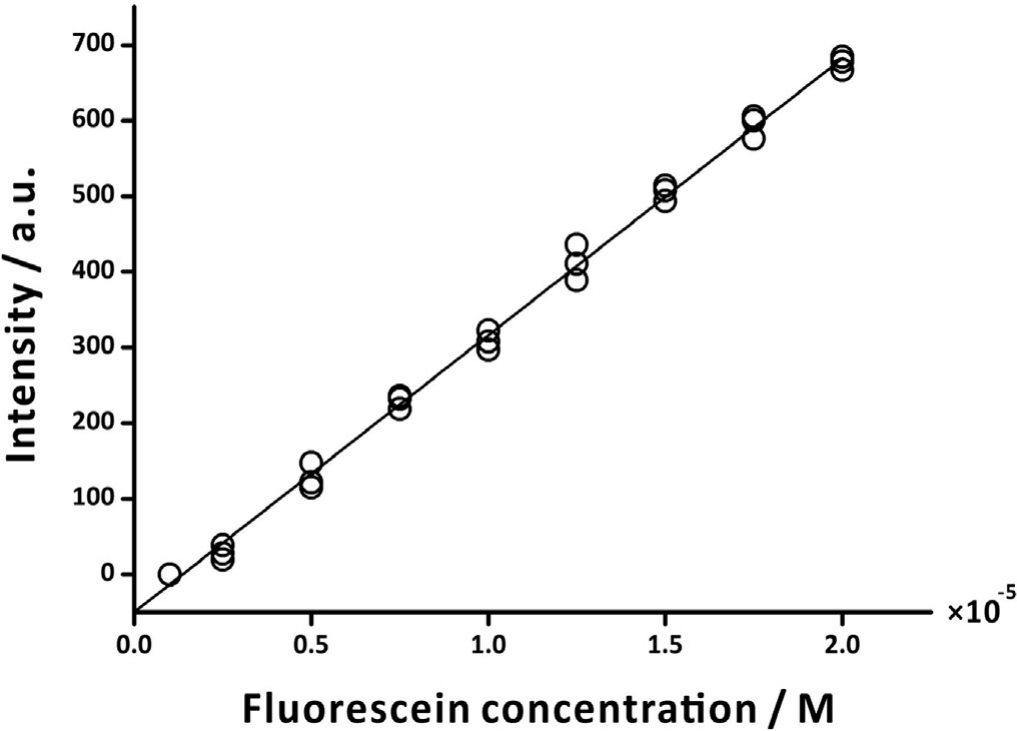
Calibration of the fluorometric probe using fluorescein solution. Calibration equation: *Intensity* = (3.66 × 10^7^ ± 4.26 × 10^5^) × *Fluorescein concentration* + (-49.81 ± 5.07); *R*^2^ = 0.99; *n* = 3. A green emission filter with transmission wavelength range 420–580 nm was used in the probe. (For interpretation of the references to colour in this figure legend, the reader is referred to the web version of this article.)

To calibrate the fluorometric probe, we prepared fluorescein solutions of concentrations in the range 1 × 10^-6^ M to 2 × 10^-5^ M in ethanol:water mixture (1:99, v/v). The pH of each solution was adjusted to 10 using sodium hydroxide (0.5 M) and hydrochloric acid (0.5 M) with the help of a pH-meter (Sartorius, Goettingen, Germany). Subsequently, to record the fluorescence intensity of the fluorescein solutions, we dipped the head of the fluorometric probe into the solutions, one after the other. During fluorescence measurement, the probe-head was immersed in the solution until the signal was stable. Then, the average value of the last five signals was plotted against fluorescein concentration. The calibration plot showed a linear trend in the tested concentration range (Fig. 8) with a limit-of-detection of 4.57 × 10^-7^ M,which is higher than the commercial fluorescence spectrometer (LS-55, PerkinElmer, Waltham, MA, USA). Nevertheless, it should be noted that, unlike in the commercial instrument, the fluorometric probe implements an inexpensive and less sensitive light detector—a photoresistor.

### 7.2 Repeatability and reproducibility of fluorescence measurements and the alcohol sensor

We characterized the fluorometric probe by testing its repeatability and reproducibility in measuring the fluorescence intensity of fluorescein solution. For the repeatability test, we prepared a fluorescein solution of concentration 1.5 × 10^-5^ M in ethanol:water mixture (1:99, v/v) and adjusted its pH to 10 using sodium hydroxide (0.5 M) and hydrochloric acid (0.5 M) with the help of a pH-meter. The fluorescence measurements were obtained using the fluorometric probe over ten times in a day to test the repeatability. A green emission filter with transmission wavelength range 420–580 nm was used in the probe. The fluorometric probe-head was dipped into the solution to take a reading and was later washed with deionized water. The repeatability was found to be 6.01% (relative standard deviation (RSD)). For the reproducibility test, we prepared fluorescein solutions (of the same concentration and pH as in the repeatability test), and measured their fluorescence using the fluorometric probe over a period of six days with three replicates per day, and the reproducibility was found to be 4.42% (RSD). Considering the 6.01% of repeatability and 4.42% of reproducibility, there is no need to recalibrate the fluorometric probe, provided the experimental conditions are same. However, for other fluorescent analytes, emission filters, or other parameters, the fluorometric probe should be calibrated once before use. Lastly, we test the repeatability of the MQ3 sensor by detecting ethanol solutions of concentration 0.2% (v/v) in water in 50-mL vials over ten times in a day. The repeatability was found to be 3.40% (RSD).

### 7.3 Measurement of fluorescence of fluorescein solutions at different pH with fluorometric probe

**Fig. 9:**
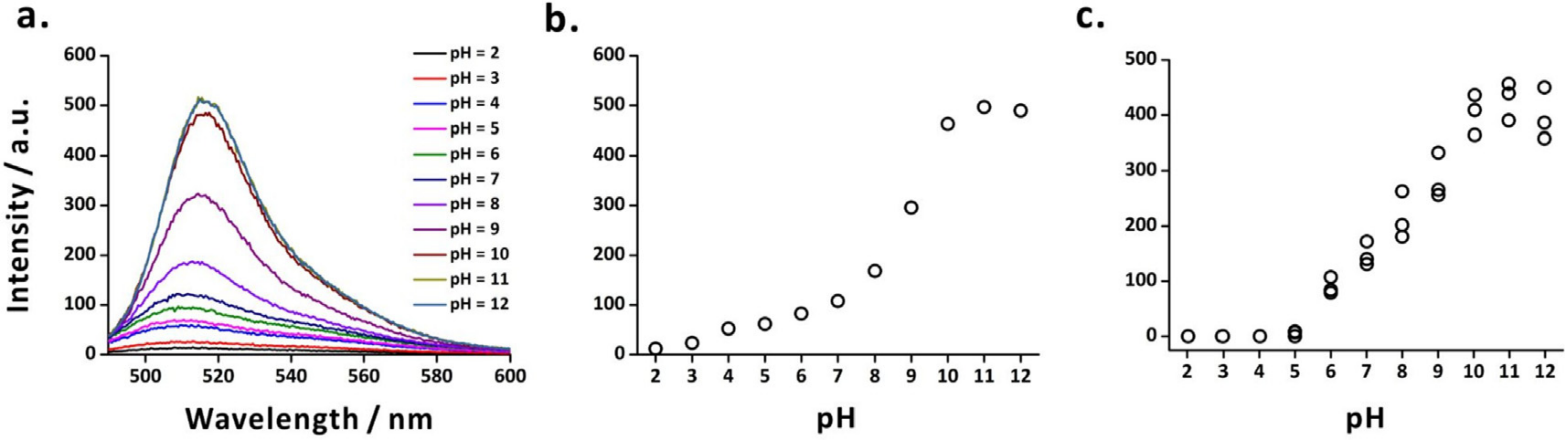
Fluorescence of fluorescein solution measured at different pH. (a) Emission spectra obtained using a commercial fluorescence spectrometer (at excitation wavelength: 470 nm), an attenuator was used to transmit only 1% of the emitted light; (b) a plot derived from (a) showing pH *vs* fluorescence intensity at emission wavelength 520 nm; and (c) fluorescence intensities measured at different pH using fluorometric probe (*n* = 3). In the probe, an LED (460–465 nm) was used as an excitation source and a green emission filter with transmission wavelength range 420–580 nm was used to transmit the emitted light. Sample: 10^-5^ M fluorescein in ethanol:water mixture (1:99, v/v). (For interpretation of the references to colour in this figure legend, the reader is referred to the web version of this article.)

To test the applicability of our fluorometric probe at different pH, we used it to measure the fluorescence of fluorescein solution (10^-5^ M) prepared in ethanol:water mixture (1:99, v/v) at different pH (from 2 to 12). The pH values were adjusted using sodium hydroxide (0.5 M) and hydrochloric acid (0.5 M) with the help of a pH-meter. To compare the performance of the probe, we also measured the fluorescence intensities of the fluorescein solutions using a fluorescence spectrometer (LS-55, PerkinElmer, Waltham, MA, USA). The emission spectra—obtained using the fluorescence spectrometer—showed a maximum emission intensity at 520 nm (Fig. 9**a**; excitation wavelength: 470 nm). The fluorescence intensities at 520 nm for the spectra obtained for fluorescein solutions at different pH shows the increase in fluorescence with increasing pH (Fig. 9**b**). The increase of pH causes conversion of the cationic form of fluorescein to the anionic forms (monoanion and dianion), resulting in strong fluorescence [37], [38]. A similar trend was observed in the fluorescence measurements obtained using the fluorometric probe (Fig. 9**c**). However, as the fluorescence intensity of fluorescein was quite faint at acidic pH (from 2 to 4), the probe could not detect the emitted light.

### 7.4 Monitoring lipase-catalyzed ester hydrolysis with fluorometric probe

Lipase-catalyzed ester hydrolysis is known to induce pH changes [39], [40]. Here, we demonstrate the use of our fluorometric probe in monitoring ester hydrolysis catalyzed by different amounts of immobilized lipase. A solution of fluorescein (10^-5^ M) was prepared in ethanol:water mixture (5:95, v/v) containing ammonium acetate (0.1 M). The initial pH of the solution was adjusted to ∼ 8 using sodium hydroxide (0.5 M) and hydrochloric acid (0.5 M) with the help of a pH-meter. Subsequently, 150 μL of propyl butyrate was added into the mixture and the reaction mixture was stirred at 400 rotations per minute (rpm) with a magnetic stirrer. Afterwards, the sensing zone of the fluorometric probe was dipped into the mixture. The fluorometric probe was placed slightly above the magnetic stir bar to avoid fluctuations in fluorescence measurements. Lastly, acrylic resin beads with immobilized lipase were added, and the reaction was monitored for 2 h.

**Fig. 10:**
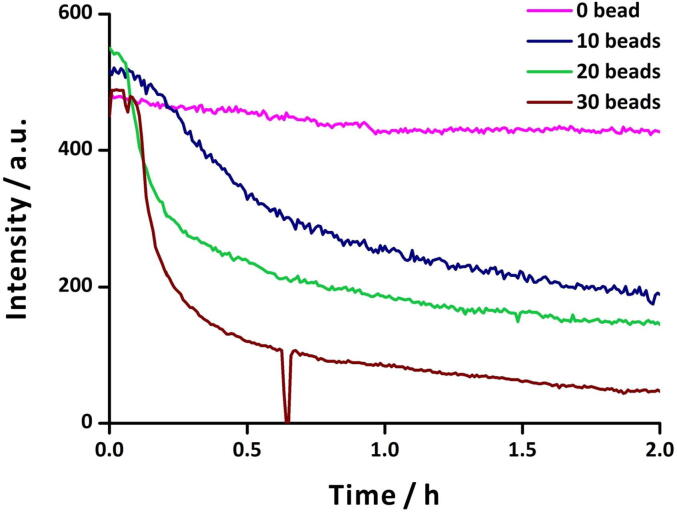
Monitoring lipase-catalyzed ester hydrolysis with fluorometric probe. Sample: a 15-mL solution of fluorescein (10^-5^ M) in ethanol:water mixture (5:95, v/v) containing ammonium acetate (0.1 M), propyl butyrate (0.067 M) and different number of acrylic resin beads (0, 10, 20, 30) with immobilized lipase. Initial pH of the sample was set to ∼ 8 in all cases. The sudden drop of fluorescence intensity at ∼ 0.7 h in the case of 30 beads is because of an abrupt shutdown of ambient light in the fume hood. A green emission filter with transmission wavelength range 420–580 nm was used in this experiment. (For interpretation of the references to colour in this figure legend, the reader is referred to the web version of this article.)

**Fig. 11:**
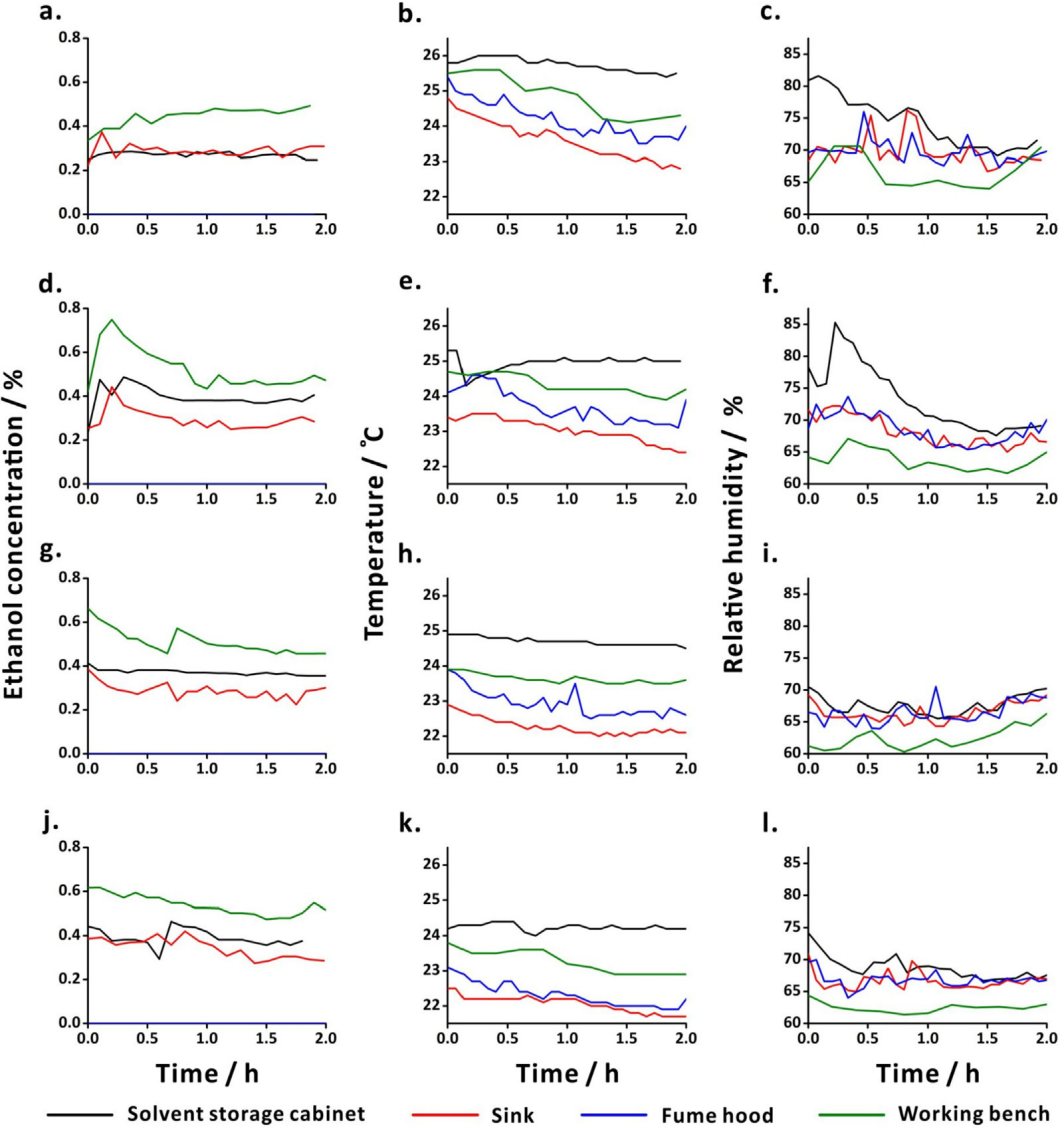
Environmental conditions at four positions in the laboratory recorded during the reaction progress of lipase-catalysed ester hydrolysis. Experiments with 0 bead (a-c); 10 beads (d-f); 20 beads (g-i); and (j-l) 30 beads of lipase. Data from alcohol sensors (a,d,g,j), temperature sensors (b,e,h,k) and humidity sensors (c,f,i,l), placed at four different positions in the laboratory (solvent storage cabinet (black line); sink (red line); fume hood (blue line); working bench (green line)). (For interpretation of the references to colour in this figure legend, the reader is referred to the web version of this article.)

We performed the ester hydrolysis reaction in the presence of different numbers of beads (0, 10, 20, and 30 beads) to observe their effect on the reaction rate (Fig. 10). In the case of 10, 20, and 30 beads, hydrolysis of propyl butyrate produces butyric acid causing significant reduction in pH. As the pH changes from basic to acidic, the fluorescence intensity of fluorescein drops, allowing one to monitor the rate of ester hydrolysis. As expected, the rate of change of pH increased with a larger number of beads of immobilized lipase. We also monitored the environmental conditions (temperature, humidity and concentration of alcohol vapor) during the progress of the reaction (Fig. 11). Although no notable influence of the ambient conditions could be observed in this experiment, the data from the environmental sensors can be helpful in experiments that are likely to be influenced by ambient conditions.

### 7.5 Monitoring synthesis of BSA-gold nanoclusters with fluorometric probe

To further validate the performance of the fluorometric probe, we planned to monitor a long-term reaction involving synthesis of gold nanoclusters (Au NCs) consisting of tens to hundreds of gold atoms that are highly fluorescent. Synthesis of Au NCs and their quantum yields are widely studied. For example, Xie *et al.* developed a green and one-pot synthetic route for Au NCs using BSA [41]. Similarly, Chang *et al.* demonstrated the synthesis of highly fluorescent gold cluster assembly with a quantum yield of up to 90% [42]. Here, we implement our fluorometric probe to monitor the synthesis of BSA-Au NCs for 12 h using the green and one-pot synthetic route developed by Xie *et al.* [41].

**Fig. 12:**
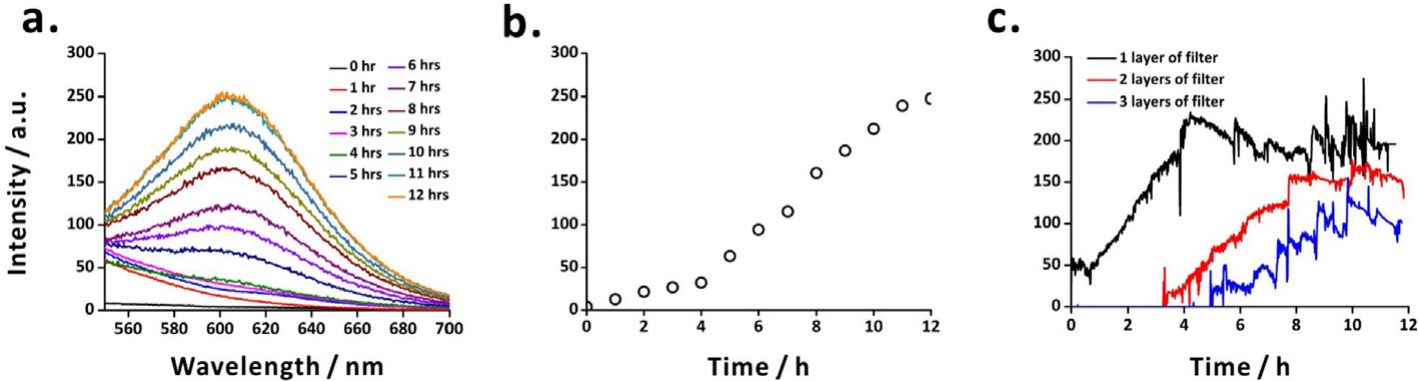
Monitoring of synthesis of BSA-Au NCs using (a) the fluorescence spectrometer (excitation wavelength: 470 nm), (b) the fluorescence intensity in different time using the fluorescence spectrometer (excitation wavelength: 470 nm; emission wavelength: 610 nm), and (c) the fluorometric probe of Telechemistry 2.0 with different amounts of layers of emission filter. One or more red emission filters with transmission wavelength range 580–700 nm were used in the probe. (For interpretation of the references to colour in this figure legend, the reader is referred to the web version of this article.)

For safety reasons, the reaction was set up in a fume hood. First, a water bath was preheated at 37 °C for 10 min. Then, 15 mL aqueous solution of hydrogen tetrachloroaurate(III) (0.01 M) and 15 mL aqueous solution of BSA (50 mg/mL) were poured into a double-necked spherical flask placed in the water bath. The reaction mixture was stirred at 500 rpm with a magnetic stirrer for 2 min. Afterwards, 1.5 mL of sodium hydroxide (1 M) solution was poured into the flask to commence the reaction. The reaction mixture was continuously stirred at 500 rpm and was incubated at 37 °C in the water bath for 12 h. To maintain a constant temperature and to avoid the evaporation of water in the flask, a layer of aluminum foil was used to cover the neck and the surface of the flask. Every hour, 1.5 mL of the reaction mixture was pipetted out and its fluorescence intensity was measured using a fluorescence spectrometer. The fluorescence intensity increased with reaction time (Fig. 12**a**) and showed highest emission intensity at wavelength 610 nm. The emission intensity at 610 nm increased slightly until 4 h, and then increased more rapidly after 4 h (Fig. 12**b**).

In a separate set of experiments, the fluorometric probe (fitted with 1, 2, or 3 layers of red emission filters) was inserted into the reaction flask through one of the necks of the flask. In the reaction mixture, the probe-head was placed slightly above the magnetic stir bar to avoid fluctuations in fluorescence measurements. The changes in fluorescence intensity were remotely monitored for 12 h *via ThingSpeak*. In the case of the probe with 1 layer of emission filter, the fluorescence intensity of the reaction mixture increased with reaction time up to about 4 h (Fig. 12**c**). However, after 4 h, the intensity either decreased or did not change much. This could be attributed to saturation of the photoresistor because the increasing trends in fluorescence intensities in the experiments with the probe fitted with 2 and 3 layers of emission filters suggest that the reaction product formed even after 4 h (Fig. 12**c**), as observed in the case of the fluorescence spectrometer (Fig. 12**a and 12b**). Notably, the fluorescence intensity was the highest in the case of the probe with 1 layer of emission filter. In the case of 2 and 3 layers of emission filters, the onset of fluorescence intensity at about 3 h and 5 h, and the decrease in intensity could be attributed to the reduced transmission of fluorescence light through the multiple layers of filter.

**Fig. 13:**
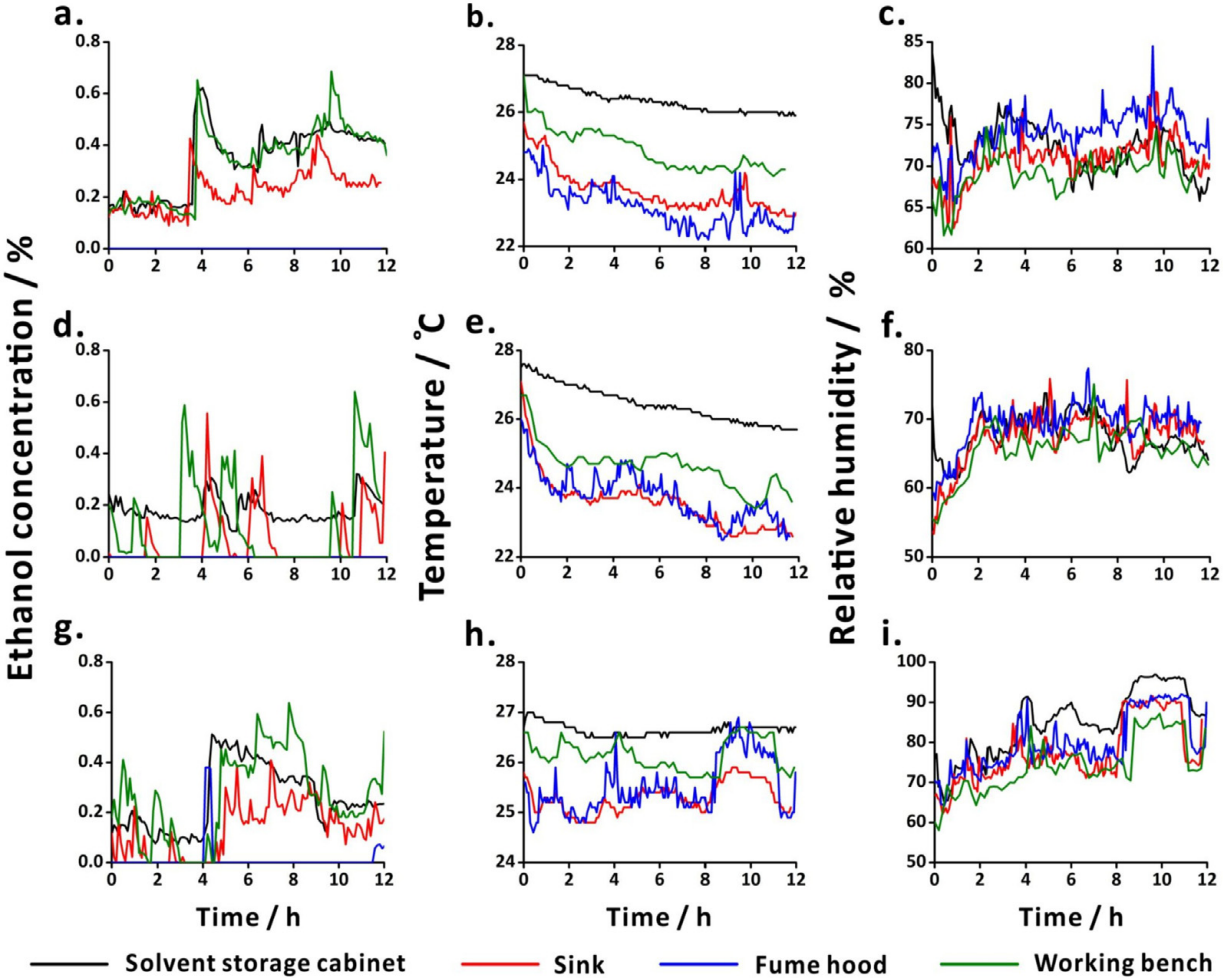
Data from the environmental sensors obtained during the reaction progress of synthesis of BSA-Au NCs. Data obtained during the experiments with the probe fitted with one layer (a-c), two layers (d-f), and three layers (g-i) of emission filters. Data from ethanol sensors (a,d,g), temperature sensors (b,e,h), and humidity sensors (c,f,i), placed at four different positions in the laboratory (solvent storage cabinet (black line); sink (red line); fume hood (blue line); working bench (green line)). (For interpretation of the references to colour in this figure legend, the reader is referred to the web version of this article.)

The environmental conditions during the synthesis of BSA-Au NCs were monitored using the auxiliary sensors (Fig. 13). Ethanol concentration in the fume hood remained 0.0% (in Fig. 13**a and 13d**) because of the continuous air flow. However, in Fig. 13**g** at about 4 h and 11 h, the ethanol concentration in the hood increased because of the use of ethanol inside the hood. Notably, the ethanol concentration dropped suddenly due to the continuous flow of air in the hood. The ambient temperature and humidity values did not show drastic changes during the experiments.

### 7.6 Concluding remarks

In this study, we present a 3D-printed, probe-type, easy-to-build fluorometric device to measure the fluorescence of a chemical reaction. The fluorometric probe can be readily fitted into a conventional reaction flask without changing the design of the flask. The sensing zone of the probe can be immersed into the reaction mixture to measure the fluorescence of the solution, like immersing a typical pH electrode in an aqueous solution to measure the pH of the solution. The probe has been fabricated using a chemically resistant material (PEEK), and therefore is compatible with common organic reaction conditions. The setup implements a Wi-Fi enabled electronic controller for real-time transmission of the experimental data to a cloud-storage platform for long-term and remote monitoring of fluorescent chemical reactions set up in a laboratory. The fluorometric device has been tested with two chemical reactions that produce green and red fluorescence. The experimental setup can be further improved by implementing a light shield around the reactor to reduce the influence of ambient light on the fluorescence measurements. Nevertheless, the fluorometric probe can be used in semi-quantitative analysis. The setup also incorporates auxiliary sensors (temperature, humidity, and alcohol vapor) to simultaneously monitor the laboratory environmental conditions, for safety purposes.

## Declaration of Competing Interest

The authors declare that they have no known competing financial interests or personal relationships that could have appeared to influence the work reported in this paper.
